# The Role of Active-Site Residues Phe98, His239, and Arg243 in DNA Binding and in the Catalysis of Human Uracil–DNA Glycosylase SMUG1

**DOI:** 10.3390/molecules24173133

**Published:** 2019-08-28

**Authors:** Danila A. Iakovlev, Irina V. Alekseeva, Yury N. Vorobjev, Nikita A. Kuznetsov, Olga S. Fedorova

**Affiliations:** 1 Institute of Chemical Biology and Fundamental Medicine (ICBFM), Siberian Branch of Russian Academy of Sciences, 8 Lavrentyev Ave., Novosibirsk 630090, Russia; 2 Department of Natural Sciences, Novosibirsk State University (NSU), 2 Pirogova St., Novosibirsk 630090, Russia

**Keywords:** DNA repair, human uracil–DNA glycosylase, SMUG1, mutant, molecular dynamics simulation, homology modeling, structure, stopped-flow kinetics, fluorescence

## Abstract

Human SMUG1 (hSMUG1) hydrolyzes the *N*-glycosidic bond of uracil and some uracil lesions formed in the course of epigenetic regulation. Despite the functional importance of hSMUG1 in the DNA repair pathway, the damage recognition mechanism has been elusive to date. In the present study, our objective was to build a model structure of the enzyme–DNA complex of wild-type hSMUG1 and several hSMUG1 mutants containing substitution F98W, H239A, or R243A. Enzymatic activity of these mutant enzymes was examined by polyacrylamide gel electrophoresis analysis of the reaction product formation and pre-steady-state analysis of DNA conformational changes during enzyme–DNA complex formation. It was shown that substitutions F98W and H239A disrupt specific contacts generated by the respective wild-type residues, namely stacking with a flipped out Ura base in the damaged base-binding pocket or electrostatic interactions with DNA in cases of Phe98 and His239, respectively. A loss of the Arg side chain in the case of R243A reduced the rate of DNA bending and increased the enzyme turnover rate, indicating facilitation of the product release step.

## 1. Introduction

Human SMUG1 (hSMUG1; single-strand selective monofunctional uracil–DNA glycosylase) is one of four human uracil–DNA glycosylases together with thymine–DNA glycosylase (hTDG), uracil–DNA glycosylase (UNG), and methyl-CpG-binding domain (hMBD4). Although all the DNA glycosylases have high affinity for uracil-containing DNA and catalyze a cleavage of the *N*-glycosidic bond involving a target base, they possess individual substrate specificity to a single-stranded DNA substrate, T/G or U/G mismatches, [1,2,3,4,5] and to some oxidized bases such as 5-hydroxymethyluracil, 5-formyluracil, and 5-hydroxyuracil, which are natural derivatives of 5-methylcytosine formed during epigenetic regulation [6].

**Figure 1:**
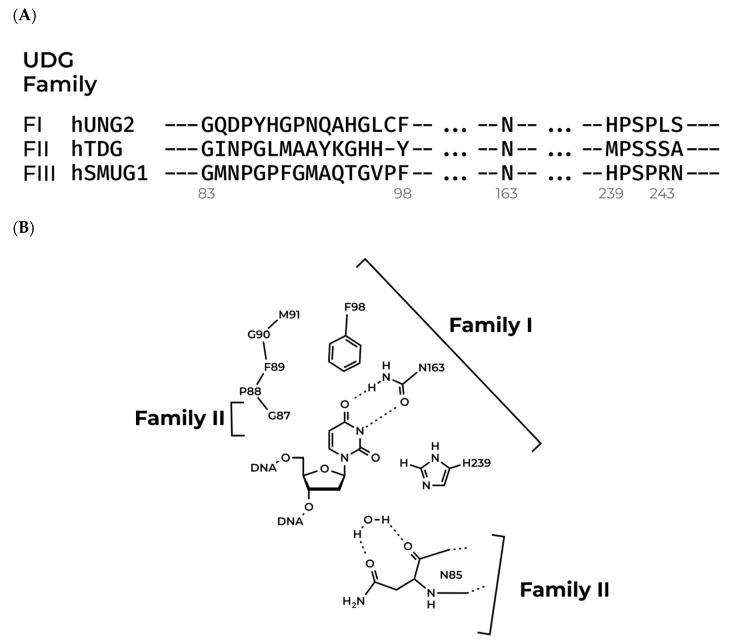
(**A**) Conserved motifs of the uracil–DNA glycosylase superfamily. (**B**) Interactions of the functionally important residues in the active-site pocket of hSMUG1, which is a hybrid of family I and II [11].

Of note, hUNG, hTDG, and hSMUG1 belong to different families (I, II, and III, respectively) of the large uracil–DNA glycosylase structural superfamily, [7] whereas hMBD4 belongs to structural superfamily HhH (helix–hairpin–helix) of DNA-binding proteins [8]. The active site of hSMUG1 represents a hybrid type, which includes the histidine-239 residue conserved in family I in the C-terminal motif and asparagine-243, which is found in family II in the N-terminal catalytic motif [7] (Figure 1A).

Although currently, the structure of hSMUG1 is unknown, the detailed structure of this enzyme was predicted [9] by homology modeling via structure-based alignment with GmeSMUG1 from *Geobacter metallireducens* (Protein Data Bank [PDB] IDs 5H98 and 5H9I) [10] and xSMUG1 from *Xenopus laevis* (PDB IDs 1OE4 and 1OE5) [2]. In structural homology and functional studies, [11] it has been reported that in hSMUG1 (Figure 1B), Asn85 and His239 catalyze the cleavage of the *N*-glycosidic bond. Asn163 and Phe98 discriminate pyrimidine bases through *π–π* stacking with Phe98 and specific hydrogen-bonding with Asn163. The Gly87–Met91 region recognizes the C5 substituent through water-bridged (uracil) or direct (hydroxy-U, hydroxymethyl-U, and formyl-U) hydrogen bonds [11]. In hSMUG1, the 239–249 sequence, called the intercalating loop, serves as a “wedge” penetrating the DNA double helix in the region of a specific site [2,11]. Amino acid residues in the intercalating loop are responsible for damaged base recognition, flipping out, and stabilization of its extrahelical state by filling the void along with the Arg243 side chain.

Pre-steady-state analysis of conformational changes of wild-type (WT) hSMUG1 and damaged DNA substrates has revealed synchronous conformational changes both in the enzyme and in the DNA [9]. It has been suggested that the initial binding of hSMUG1 to DNA leads to a movement of the intercalating loop of the enzyme and local DNA melting near the specific site, in agreement with the “wedge” strategy of damage search by DNA glycosylases of different structural families [12,13,14,15,16,17].

To verify the damage recognition process, in the present study, we modeled the structures of hSMUG1–DNA complexes with the WT enzyme and a few mutant forms of the enzyme containing substitution of catalytic amino acid residue His239 with Ala, a substitution of Phe98, which directly interacts with the damaged base, with Trp or a substitution of Arg243 in the intercalating loop with Ala. The conformational dynamics of the enzymes and DNA were directly recorded by the stopped-flow technique combined with fluorescence detection. A comparison of fluorescence kinetic data obtained allowed us to elucidate the roles of Phe98, His239, and Arg243 in transient stages of specific and nonspecific DNA binding, in damaged base recognition, and in catalysis.

## 2. Materials and Methods

### 2.1. Model Structure of hSMUG1

A homology model of hSMUG1 was obtained previously [9] and used as a template for further modeling of WT hSMUG1 and of its mutant versions. First, the WT hSMUG1 model was modified to determine the initial structures of mutants F98W, H239A, and R243A in the Chimera software (ver. 1.13.1, University of California, San Francisco, CA, USA). Each amino acid substitution was performed by the removal of the side chain of a residue being studied from the original PDB file and via reconstruction of a new side chain from a rotamer library [18].

Initial models were then optimized by molecular dynamics (MD) simulation using a modified AMBER ff99 force field [19,20,21] and the BioPASED [21] front-end program. Proteins were equilibrated at 50 K using the implicit Gaussian water model [22] and next annealed to 300 K for 100 ps. Production simulations were performed for 5 ns, both potential energy and overall root mean square deviation (RMSD) were stable during the simulation. Representative structures of all trajectories (centroids in pairwise all-atom-RMSD space) served as templates for further investigation.

### 2.2. Modeling the Structure of the Enzyme–DNA Complex

Given that there are no crystal structures of UDG family III enzymes in complex with double-stranded DNA, we expanded our search to the whole UDG superfamily to find a properly organized enzyme–DNA complex to predict the structure of hSMUG1 bound to DNA.

The conserved domain database [23] was screened to find all structures of protein–DNA complexes that belong to the UDG superfamily. After that, the obtained structures were structurally aligned by means of the Chimera “Match maker” tool to match proteins (Needleman–Wunsch algorithm, BLOSUM-62 matrix, default secondary structure-scoring parameters) and then DNAs (Needleman–Wunsch algorithm, nucleic-acid matrix). The homology model of WT human SMUG1 described previously [9] was chosen as a template (Figure S1). Structural alignment with attention to overlaps in the damaged base-binding pocket, intercalating loop, and overall, in the protein–DNA interface revealed that the X-ray structure of the hTDG–DNA complex (PDB ID: 5T2W) fits the model hSMUG1 structure the best. Therefore, the initial structure of the WT hSMUG1–DNA complex was produced by combining model hSMUG1 with DNA taken from the X-ray structure of the hTDG–DNA complex (PDB ID: 5T2W).

The DNA duplex from 5T2W was truncated and modified to obtain the 17-mer double-stranded DNA (U-substrate) that served as a substrate in this work (see Table 1).

The initial complex was optimized for 10 ns following a simulation protocol for the free enzyme. The structure of complex WT hSMUG1–DNA was then modified to obtain structures of complexes of hSMUG1 mutants F98W, H239A, and R243A with DNA. The structures of the mutants bound to DNA were next optimized for 10 ns by the same protocol, and representative structures were then analyzed.

Close-up views of the structures were prepared using Chimera [24] and Blender software (ver. 2.79, Stichting Blender Foundation, Amsterdam, The Netherlands).

### 2.3. Protein Expression and Purification

The full-size proteins WT hSMUG1 and mutants F98W, H239A, and R243A were isolated from *Escherichia coli* Rosetta 2 cells transformed with respective plasmids. Cells of *E. coli* Rosetta 2 were cultivated in 2 × YT medium (1 L) containing 50 μg/mL kanamycin at 37 °C until absorbance at 600 nm (A_600_) of 0.6 to 0.7. After that, the temperature was lowered to 20 °C, and transcription was induced by adding isopropyl-β-d-thiogalactopyranoside to 0.2 mM for overnight incubation.

After that, the cells were incubated for 16 h and then centrifuged (relative centrifugal force 9000× *g*, 10 min). A cell suspension was prepared in 40 mL of lysis buffer (20 mM HEPES-NaOH pH 7.8 and 40 mM NaCl) with the addition of a protease inhibitor cocktail (cOmplete, Roche Diagnostics, Mannheim, Germany). The cells were lysed in a Thermo French Pressure Cell Press.

All the subsequent procedures were conducted at 4 °C. Each cell lysate was centrifuged at relative centrifugal force 40,000× *g* for 40 min. Next, 1 M NaCl was added to the supernatant to 100 mM concentration. Then, the supernatant was loaded onto an equilibrated column I (V = 30 mL, Q-Sepharose Fast Flow, Amersham Biosciences, Sweden) with subsequent washing with buffer solution I (20 mM HEPES-NaOH pH 7.8 and 100 mM NaCl).

The fraction containing the enzyme was collected and loaded onto an equilibrated column II (HiTrap-Chelating™, Amersham Biosciences, Uppsala, Sweden) in buffer solution II (20 mM HEPES-NaOH pH 7.8, 500 mM NaCl, and 20 mM imidazole). Chromatography was run in buffer solution II and a linear gradient of 20 → 500 mM imidazole. Absorbance of the solution was measured at a wavelength of 280 nm.

Protein purity was determined by the Laemmli polyacrylamide gel electrophoresis (PAGE) protocol. Fractions containing the hSMUG1 protein were diluted two-fold with glycerol and stored at −20 °C. The protein concentration was calculated by the Bradford method. All the experiments on the enzymatic reaction were conducted at 25 °C in a buffer consisting of 50 mM Tris-HCl pH 7.5, 50 mM KCl, 1 mM EDTA, 1 mM dithiothreitol (DTT), and 7% (*v*/*v*) glycerol.

### 2.4. Oligodeoxynucleotides (ODNs)

The sequences of the DNA substrates employed in this work are presented in Table 1. The ODNs were synthesized via the standard phosphoramidite method on an ASM-700 synthesizer (BIOSSET Ltd., Novosibirsk, Russia) using phosphoramidites purchased from Glen Research (Sterling, VA, USA). The synthetic ODN was uncoupled from the solid support with an ammonia solution according to the manufacturer’s protocol.

The deprotected ODNs were purified by high-performance liquid chromatography. The purity of ODNs exceeded 98% as estimated by electrophoresis in a 20% polyacrylamide gel after staining with Stains-All dye (Sigma-Aldrich, St. Louis, MO, USA). Concentrations of the nucleotides were determined by a light absorption assay at A_260_.

The ODN duplexes were prepared by annealing modified and complementary strands in a 1:1 molar ratio in a buffer consisting of 50 mM Tris-HCl pH 7.5, 50 mM KCl, 1 mM EDTA, 1 mM DTT, and 7% (*v*/*v*) glycerol.

### 2.5. PAGE Analysis of Time-Course Experiments

The reaction mixture contained 5.0 μM U-substrate, 1.0 μM hSMUG1 (WT, F98W, H239A, or R243A), 50 mM Tris-HCl pH 7.5, 50 mM KCl, 1.0 mM EDTA, 1.0 mM DTT, and 7% (*v*/*v*) glycerol. The damaged strand of U-substrate was 5′-labeled using phage T4 polynucleotide kinase (New England Biolabs, Beverly, MA, USA) and [γ^32^P]ATP (4500 Ci/mol). The labeled ODN was purified on Sephadex G-25 columns (GE Healthcare, Little Chalfont, UK).

The reaction was initiated by the addition of the enzyme. Aliquots (3.5 μL) of the reaction mixture were withdrawn and immediately quenched with 3.5 μL of a gel-loading dye solution consisting of 7 M urea with 0.1% (*w*/*v*) bromophenol blue and 0.1% (*w*/*v*) xylene cyanol. After that, 3.0 mL of a 33% aqueous piperidine solution was added with incubation at 37 °C for 30 min. Piperidine treatment was performed on all samples before gel-loading (including time point zero) and resulted in complete cleavage of DNA substrates at AP-sites generated by hSMUG1.

Aliquots were then precipitated by the addition of a 10-fold volume of a 2% LiClO_4_ solution in acetone; the pellet was washed with acetone and dissolved in 5 μL of the gel-loading dye. The solution was loaded on a 20% (*w*/*v*) polyacrylamide/7 M urea gel. Conversion of the substrate to the product was analyzed by autoradiography on a Phosphor Imager and quantified by scanning densitometry in the Gel-Pro Analyzer software, v. 4.0 (Media Cybernetics, Rockville, MD, USA). The measurement error usually did not exceed 20%.

The observed rate constants were estimated according to exponential Equation (1).

[*Product*] = *A* × (1 − exp(−*k* × *t*)) (1)

### 2.6. Circular Dichroism (CD) Spectra

These spectra were recorded on a Jasco J-600 spectropolarimeter (Jasco, Tokyo, Japan), at 5 °C in quartz cells with 1-cm path length. The spectra were recorded at the bandwidth 1.0 nm and resolution 1.0 nm with a scan speed of 50 nm/min. The scans were accumulated and automatically averaged. The concentration of enzyme in the cell was 1.0 µM. The experiments were carried out in the buffer consisting of 50 mM Tris-HCl pH 7.5, 50 mM KCl, 1.0 mM EDTA and 7% glycerol (*v*/*v*). Because of aggregation of F98W hSMUG1 under this condition for this mutant form CD spectrum was recorded in high salt concentration that increases the noise up to 210 nm in the spectrum.

### 2.7. Stopped-Flow Fluorescence Kinetics

The activity of WT hSMUG1 and that of the mutants were analyzed via recording of fluorescence kinetics as described elsewhere [25,26,27,28]. In brief, we used an SX.20 stopped-flow spectrometer (Applied Photophysics Ltd., Leatherhead, UK) equipped with a 150 W Xe arc lamp and an optical cell with 2 mm path length. The dead time of the instrument is 1.4 ms. If a 6-carboxyfluorescein (FAM) fluorophore was present in the ODN duplex (X-FRET-substrates), the wavelength λ_ex_ = 494 nm was used to excite the fluorescence of these residues, and their emission was analyzed at λ_em_ = 515 nm (Schott filter OG-515).

All the experiments were conducted at 25 °C in a buffer consisting of 50 mM Tris-HCl pH 7.5, 50 mM KCl, 1.0 mM EDTA, 1.0 mM DTT, and 7% (*v*/*v*) glycerol. In a standard procedure, the solution of hSMUG1 was placed in one instrument’s syringe and rapidly mixed in the reaction chamber with the substrate from another syringe. The reported concentrations of reactants are concentrations in the reaction chamber after mixing. Typically, each trace depicted in the figures is the average of four or more fluorescence traces recorded in individual experiments. In the figures, if necessary for better presentation, the curves were manually moved apart. This procedure does not affect the results of fitting because the background fluorescence is fitted separately for each curve.

### 2.8. Kinetic Data Analysis

Stopped-flow kinetic traces were fitted to Equation (2) by a nonlinear regression procedure in the Origin software (OriginLab Corp., Northampton, MA, USA):

(2) $$F_{c}=F_{b}+\sum_{i=0}^{N}A_{i}\times exp\;(-k_{i}\times t)$$

where *F* is the FRET signal, *F_b_* indicates background fluorescence, *A_i_* denotes fluorescence parameters, *k*_i_ is the observed rate constant, and *t* is the reaction time.

## 3. Results and Discussion

### 3.1. Modeling of the Structures of hSMUG1 Complexes with a DNA Substrate

Because the structures of human SMUG1 have not yet been obtained yet, the MD simulation approach was chosen to model the structures of free WT hSMUG1 and free mutants F98W, H239A, and R243A as well as their complexes with DNA. All obtained structures were stable during 10 ns in the MD simulation and shared an identical fold, indicating that a single amino acid substitution did not disturb the global protein structure and protein–DNA interface.

**Figure 2:**
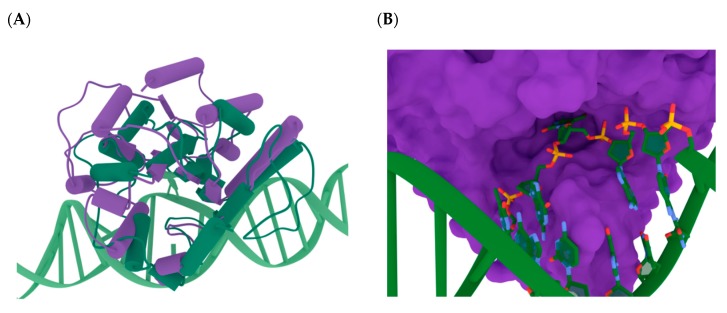
(**A**) Structural alignment of hTDG (PDB ID 5T2W, green) and hSMUG1 (model, purple) with DNA. Protein α-helices (pipes) and β-strands (planks) and DNA coil ribbons are shown. (**B**) Combination of the DNA from the 5T2W structure with the solvent-accessible surface of hSMUG1. The uracil base was found to be snugly located in the hSMUG1 active-site pocket. The intercalating loop of hSMUG1 precisely fitted into the appropriate void of DNA.

Alignment of the model structure of free hSMUG1 with all structures of the enzymes belonging to the UDG superfamily revealed that hTDG in complex with DNA matches WT hSMUG1 well (Figure 2A). Of note, functionally important regions of the hSMUG1–DNA complex such as the active site, intercalating loop, and overall, the protein–DNA interface (Figure S2) were carefully analyzed to reveal structural similarities. Indeed, common major features of the protein–DNA interface, hydrophobic damaged base-binding pocket, and intercalating loop aligned perfectly despite poor global homology (16.6%) between hTDG and hSMUG1 (Figure 2B).

**Figure 3:**
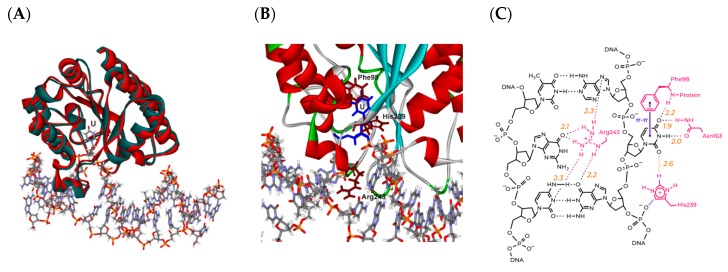
(**A**) An overlay of the structures of free hSMUG1 (red) and of hSMUG1 complexed with DNA (green). The Ura base (U) located in the active-site pocket is also shown. (**B**) Positions of the studied amino acid residues Phe98, His239, and Arg243 in the hSMUG1–DNA complex. (**C**) Schematic representation of contacts formed by Phe98, His239, and Arg243 in the complex of hSMUG1 with DNA.

By means of the DNA coordinates from the structure of complex hTDG–DNA (PDB ID: 5T2W), the structure of the WT hSMUG1–DNA complex was optimized by MD simulation. As presented in Figure 3A, the formation of a complex with DNA does not lead to significant structural rearrangement of the hSMUG1 molecule during the simulation period. Nevertheless, side chains of functionally important amino acid residues Phe98, His239, and Arg243 adjusted to accommodate the everted Ura base (Figure 3B) and to form an extensive protein–DNA intermolecular contacts (Figure 3C). Phe98 is involved in the stacking interaction with the flipped out Ura base thus stabilizing it in the base-binding pocket of the active site. His239 handles the DNA backbone via an electrostatic contact with the phosphate group, and Arg243 gets inserted into the void formed after Ura base flipping out from the DNA duplex and forms a large network of hydrogen bonds with neighboring nucleobases.

**Figure 4:**
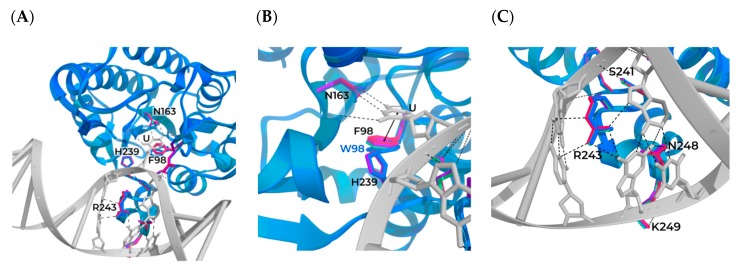
Structural alignment of WT hSMUG1 and of mutants. (**A**) Superposition of WT hSMUG1 (magenta), F98W (blue), H239A (cyan), and R243A (violet) structures in complex with DNA. Close-up view of the locations of Phe98 and His239 (**B**) and hydrogen bonds formed by Arg243 with DNA (**C**) in the active site of the enzyme. Direct contacts are shown as dashed black lines.

The model structure of the hSMUG1–DNA complex was employed for in silico substitution of Phe98, His239, or Arg243 to build the structures of mutants F98W, H239A, and R243A (Figure 4). It was demonstrated that the substitution of Phe98 with Trp leads to disruption of the stacking with the Ura base thereby blocking the formation of a catalytically competent conformation of the active site (Figure 4B). This finding explains the reduced activity of the F98W mutant in comparison with WT hSMUG1. On the other hand, mutation H239A did not cause a significant perturbation of the protein–DNA network contacts. Nevertheless, the H239A mutant is catalytically inactive due to the absence of the imidazole residue essential for hydrogen-bonding with the phosphate group. According to the MD simulation, protein–DNA contacts were not disturbed by substitution R243A either (Figure 4C). These data indicated that even though Arg243 forms numerous hydrogen bonds with nucleobases, this amino acid residue is not the key residue participating in stabilization of the extrahelical state of the Ura base. Nonetheless, Arg243 may be important for the initial step of recognition of damage in the DNA chain.

### 3.2. CD Analysis of WT hSMUG1 and of the Mutants

**Figure 5:**
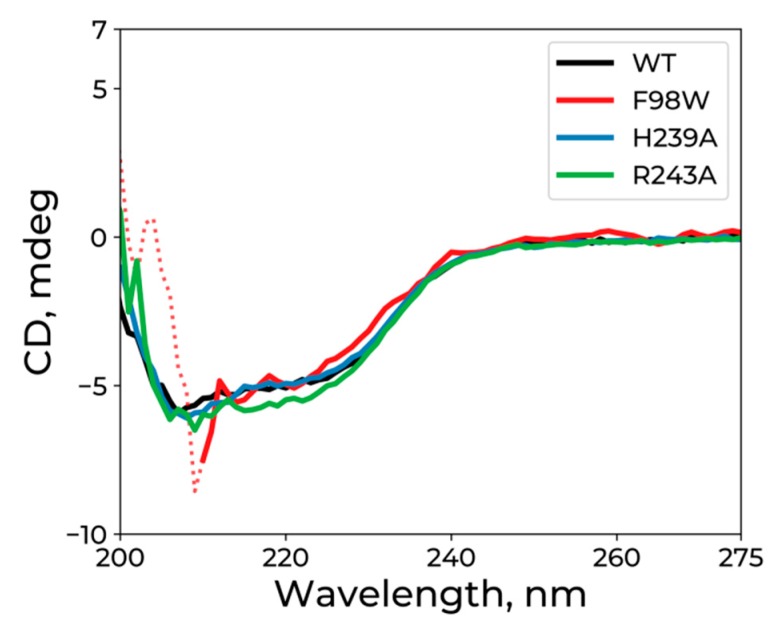
CD spectra of WT hSMUG1 and mutants F98W, H239A, and R243A. A high noise up to 210 nm in the spectrum of F98W (dashed curve) was due to high salt concentration in the sample (see Materials and Methods).

CD experiments were conducted to determine the extent of changes in the conformations of WT hSMUG1 and mutants F98W, H239A, and R243A in a free state (Figure 5). The CD spectra of all the mutants contained a wide negative band from 208 to 225 nm, which is an indicator of the α-helical structure of a protein. CD spectra of the WT enzyme and mutants have close shapes, suggesting that the mutations do not induce global structural rearrangements of the enzyme.

### 3.3. Relative N-Glycosylase Activity of WT hSMUG1 and of the Mutants

**Figure 6:**
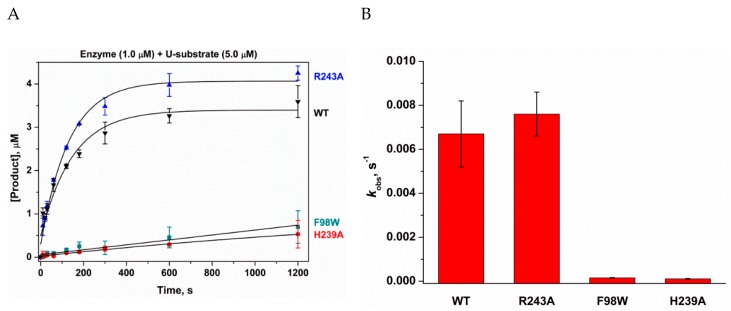
Accumulation of the reaction product (**A**) and relative activity (**B**) of WT hSMUG1 and mutants F98W, H239A, and R243A during the interaction with the model DNA duplex containing a U/G pair as determined by PAGE. [Enzyme] = 1.0 × 10^−6^ M, [Substrate] = 5.0 × 10^−6^ M.

To estimate the enzymatic activity of the mutant hSMUG1 enzymes, the kinetics of product formation were studied by PAGE (Figure 6A). The observed rate constants were estimated according to exponential Equation (1) (Figure 6B). The mutation of catalytically important amino acid residue His239 or a substitution of Phe98 (which stacks with the Ura base in the base-binding pocket) resulted in a significant loss of the enzymatic activity. In contrast, it was found that the activity of R243A hSMUG1 was slightly higher in comparison with the WT enzyme. Elsewhere, it has been shown that hSMUG1 binds tightly to product AP-sites, [1,11,29] and thus the product release could be a rate-limiting step of the enzyme turnover [9]. Therefore, the obtained data on relative activity indicate that the R243A substitution facilitates product dissociation, probably due to the disruption of numerous hydrogen bonds formed by the Arg243 side chain with DNA (Figure 3C).

### 3.4. Pre-Steady-State Analysis of Conformational Dynamics of WT hSMUG1 and of the Mutants

**Scheme 1:**
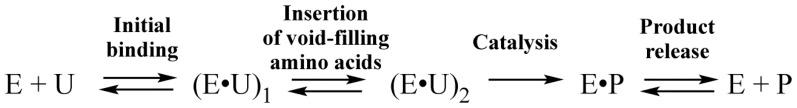
A summary kinetic scheme describing the interaction of WT hSMUG1 with DNA as revealed by different fluorescent reporters [9].

Previously, [9] on the basis of combination of the fluorescence data reflecting the conformational transitions in WT hSMUG1 (Trp) and in DNA (2-aminopurine and FRET) in the course of the catalytic cycle, a stepwise mechanism of specific DNA binding and catalysis was suggested (Scheme 1). Step 1 in this scheme was attributed to the initial DNA binding involving a movement of the intercalating loop of the enzyme, DNA melting in the vicinity of the specific site, and possibly Ura base eversion from DNA; step 2 consists of insertion of void-filling amino acids into DNA; step 3 is hydrolysis of the *N*-glycosidic bond; and step 4 represents a slow process of the product release accompanied by a DNA conformational change.

Where E is hSMUG1; U is the U-substrate; (E•U)_1_ denotes one of enzyme–substrate complexes; P is the product of the *N*-glycosylase reaction.

**Figure 7:**
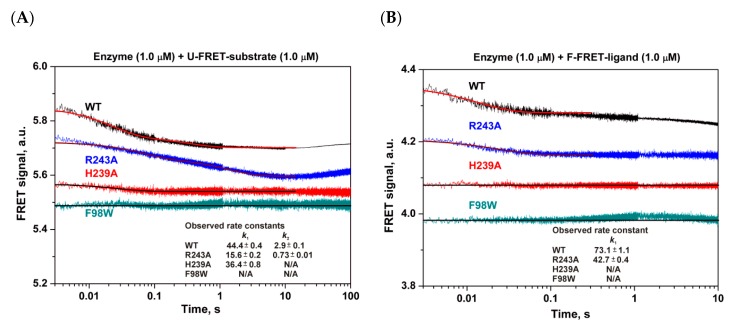
The interaction of WT hSMUG1 or of the mutants with DNA duplexes containing a Ura base (**A**) or F-site (**B**). Concentrations of the enzymes and DNA are indicated in the panels.

The interactions of WT hSMUG1 or of the mutants with DNA duplexes containing an Ura base or a stable abasic-site analog [(2*R*,3*S*)-2-(hydroxymethyl)-3-hydroxytetrahydrofuran: F-site] were recorded by means of changes in the FRET signal (Figure 7). As suggested earlier regarding the WT enzyme [9], the initial decrease in the FRET signal (0.005–0.100 s) reflects the shortening of the distance between FAM and BHQ1, probably owing to DNA bending. The second decrease phase of the FRET signal proceeding slowly in a time range of 0.1–10 s uncovered an additional conformational adjustment of DNA in complex with the enzyme. Exponential fit of these curves allowed to calculate observed rate constants of the first and the second phases, if applicable.

It is worth noting that the two mutants with the reduced catalytic activity (F98W and H239A) did not yield any FRET signal changes during the interaction with DNA. These data support the conclusion that in both cases, the bending of DNA during the formation of a catalytically competent conformation of the enzyme is blocked by disruption of specific contacts, which may be generated by these amino acid residues and DNA at the step of initial binding (step 1).

Interaction of R243A hSMUG1 with the U-FRET-substrate shows one decrease in intensity up to 10 s. Nevertheless, this curve was best fitted by double exponential equation. Substitution R243A reduced the observed rate constant *k*_1_ of DNA bending in 2.8-fold as well as reduced the observed rate constant *k*_2_ in 4-fold, which characterizes additional adjustment of DNA in complex with the enzyme (Figure 7A). In the case of interaction R243A hSMUG1 with F-FRET-ligand, the time-course of the FRET signal features one visible decrease of intensity in the initial period (up to 1 s) well matching the shape of the kinetic curve for the WT enzyme (Figure 7B). However, again the observed rate constant of decrease phase was 1.7-fold lesser in the case of R243A hSMUG1 if compare with WT enzyme. Although R243A hSMUG1 contains a methyl group instead of the arginine side chain, the void is still filled with other amino acid residues of the intercalating loop, namely Ala243 and main chains of Asn244 and Pro245, thus causing the bending of conformationally flexible abasic DNA. In the case of the more conformationally stable DNA duplex containing Ura, the absence of specific contacts formed by Arg243 leads to a reduction in the observed rate of DNA bending up to 10 s (Figure 7A). Probably, this disturbance leads to an increase in the rate of catalytic-complex [Scheme 1, complex (E•U)_2_] disruption in both directions: to initial complex (E•U)_1_ or, if the catalytic step was reached, to facilitate a release of the product.

## 4. Conclusions

The model structure of a complex of hSMUG1 with damaged DNA was constructed and optimized by MD simulations. The structure revealed that hSMUG1 forms an extensive network of contacts with DNA in the catalytically active complex involving certain amino acid residues. Phe98 is involved in the stacking interaction with the flipped out Ura base, thereby stabilizing it in the base-binding pocket of the active site. His239 handles the DNA backbone via an electrostatic contact with a phosphate group, and Arg243 gets inserted into the void formed after Ura base flipping out from the DNA duplex and forms a large network of hydrogen bonds with neighboring nucleobases. To examine the functions of these amino acid residues, hSMUG1 mutants F98W, H239A, and R243A were used with estimation of their enzymatic activity by PAGE analysis of the reaction product formation and by pre-steady-state analysis of DNA conformational changes in the course of the binding of FRET-labeled DNA substrates. It was shown that substitutions F98W and H239A lead to a loss of the catalytic activity, probably owing to a disruption of specific contacts generated by these residues. This situation prevents DNA bending at the initial steps of complex formation. On the other hand, mutation R243A diminishes the rate of DNA bending and increases the enzyme turnover rate, thus pointing to facilitation of the product release step.

## Figures and Tables

**Table 1:** DNA duplexes used as substrates and ligands of hSMUG1 ^a^.

*Shorthand*	*Sequence*
U-substrate	5′-GCTCA**U**GTACAGAGCTG-3′ 3′-CGAGTGCATGTCTCGAC-5′
F-ligand	5′-GCTCA**F**GTACAGAGCTG-3′ 3′-CGAGTGCATGTCTCGAC-5′
U-FRET-substrate	5′-**FAM**-GCTCA**U**GTACAGAGCTG-3′ 3′-CGAGTGCATGTCTCGAC-**BHQ1**-5′
F-FRET-ligand	5′-**FAM**-GCTCA**F**GTACAGAGCTG-3′ 3′-CGAGTGCATGTCTCGAC-**BHQ1**-5′

^a^ BHQ1 is black hole quencher 1, F is (2*R*,3*S*)-2-(hydroxymethyl)-3-hydroxytetrahydrofuran, FAM is 6-carboxyfluorescein, and FRET is Förster resonance energy transfer.

## Supplementary Materials

The following are available online. Figure S1: Structural alignment of UNG superfamily proteins (green, blue, purple, pipes and planks) with DNA (magenta ribbons). Figure S2: Functionally important regions on enzyme surface: active site (red), protein-DNA interface (yellow), intercalating loop (blue).

## Abbreviations

SMUG1	single-stranded selective monofunctional uracil–DNA glycosylase;
AP-site	apurinic/apyrimidinic site;
F-site	(2*R*,3*S*)-2-(hydroxymethyl)-3-hydroxytetrahydrofuran residue;
ODN	oligodeoxyribonucleotide;
PAGE	polyacrylamide gel electrophoresis;
BER	base excision repair;
FRET	Förster resonance energy transfer;
AMBER	Assisted Model Building with Energy Refinement.
