# Supplementary material for: The Role of Active-Site Residues Phe98, His239, and Arg243 in DNA Binding and in the Catalysis of Human Uracil–DNA Glycosylase SMUG1
Source: Molecules. 2019 Aug 28;24(17):3133. doi: 10.3390/molecules24173133 (PMC6749576; doi:10.3390/molecules24173133)
Supplement: Supplementary file 1 [file molecules-24-03133-s001.pdf]

The role of active-site residues Phe98, His239, and Arg243 in DNA binding and in the catalysis of human uracil-DNA glycosylase SMUG1

Danila A. Iakovlev[1], Irina V. Alekseeva[1], Nikita A. Kuznetsov*[1,2] and Olga S. Fedorova*[1,2]

[a] Institute of Chemical Biology and Fundamental Medicine (ICBFM), Siberian Branch of Russian Academy of Sciences, 8 Lavrentyev Ave., Novosibirsk 630090, Russia

[b] Department of Natural Sciences, Novosibirsk State University (NSU), 2 Pirogova St., Novosibirsk 630090, Russia

*To whom correspondence should be addressed. O.S.F. (Tel.: +7 383-3635175, Fax: +7 383-3635153, e-mail: fedorova@niboch.nsc.ru), N.A.K. (Tel.: +7 383-3635174, Fax: +7 383-3635153, e-mail: nikita.kuznetsov@niboch.nsc.ru).

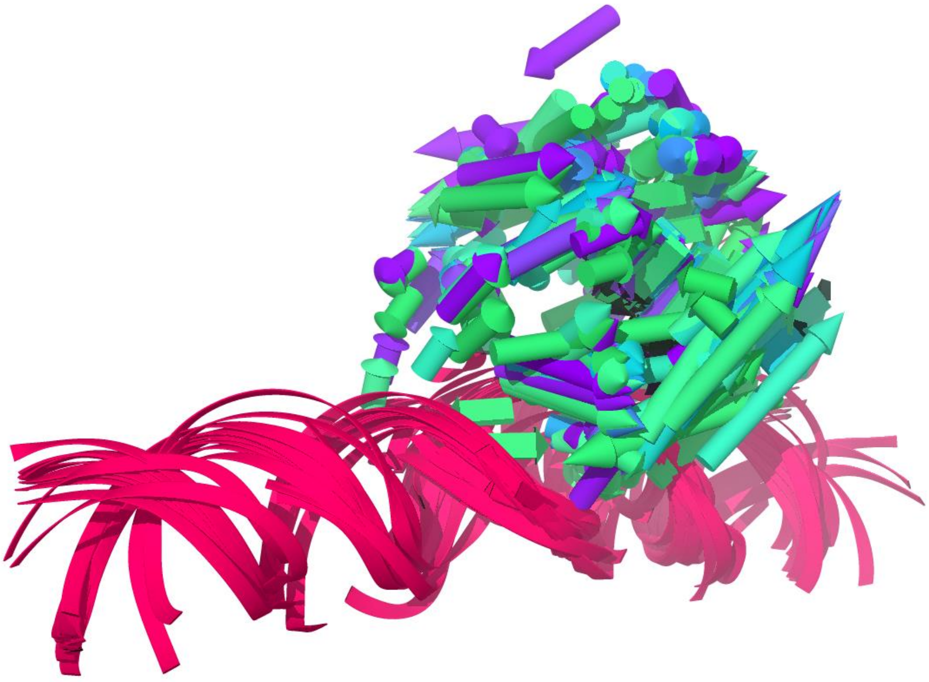

Supplementary Figure S1. Structural alignment of UNG superfamily proteins (green-blue-purple pipes and planks) with DNA (magenta ribbons).

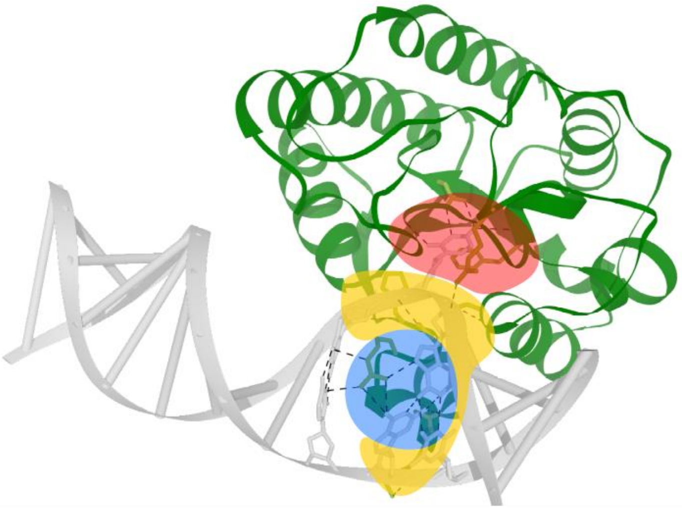

Supplementary Figure S2. Functionally important regions on enzyme surface: active site (red), protein-DNA interface (yellow), intercalating loop (blue).
